# Selfies and social media: relationships between self-image editing and photo-investment and body dissatisfaction and dietary restraint

**DOI:** 10.1186/2050-2974-3-S1-O21

**Published:** 2015-11-23

**Authors:** Siân A McLean, Susan J Paxton, Eleanor H Wertheim, Jennifer Masters

**Affiliations:** 1 School of Psychology and Public Health, La Trobe University, Australia; 2 School of Education, La Trobe University, Australia

## 

Engagement with social media, such as Facebook, Instagram, and Snapchat, may lead to negative outcomes for body dissatisfaction and disordered eating due to the appearance focused nature of the online interactions. The aim of this cross-sectional study was to examine relationships between social media photo-related activities and overvaluation of shape and weight, body dissatisfaction, and dietary restraint in adolescent girls. Participants were 101 year 7 girls (Mage = 13.1, SD = 0.3) who completed measures of social media use and body image and disordered eating via self-report. Significantly higher levels of overvaluation of shape and weight, body dissatisfaction, dietary restraint, and internalisation of the thin ideal were found for participants who regularly shared self-images on social media, compared with those who were not regular sharers. In addition, after controlling for media exposure and internalisation of the thin ideal, higher engagement in manipulation of self-images and greater investment in the self-images prior to sharing were associated with greater overvaluation of shape and weight, body dissatisfaction, and dietary restraint. Findings suggest that self-image related social media activities may contribute to body dissatisfaction and disordered eating and indicate important contemporary targets for social media based intervention for these problems.

